# Myelomatous Pleural Effusion as a Presenting Symptom: A Case Report

**DOI:** 10.7759/cureus.5153

**Published:** 2019-07-16

**Authors:** Alejandro Rodelo, Jitesh Joshi

**Affiliations:** 1 Medical Oncology / Hematology, Houston Methodist Hospital, Houston, USA

**Keywords:** multiple myeloma, pleural effusion, hematology

## Abstract

Myelomatous pleural effusion (MPE) is a rare complication in patients suffering from multiple myeloma (MM). Dyspnea is the predominant symptom in the clinical presentation. Poor prognosis and aggressive MM behavior have been linked to this condition. We describe a case of MPE in an undiagnosed MM patient who presented with respiratory discomfort and general malaise.

## Introduction

Multiple myeloma (MM) is a malignant plasma cell disorder accounting for 10% of all hematologic malignancies [1]. Hallmarks for this disease can be summarized by the CRAB mnemonic: hypercalcemia, renal failure, anemia, and lytic bone lesions predominantly of the skull [2]. MM is theorized to evolve from an asymptomatic premalignant stage known as monoclonal gammopathy of undetermined significance (MGUS) with a 1% yearly risk of progression [1]. We report a case of a patient presenting with MM and pleural effusion as her initial symptom.

## Case presentation

**Figure 1:**
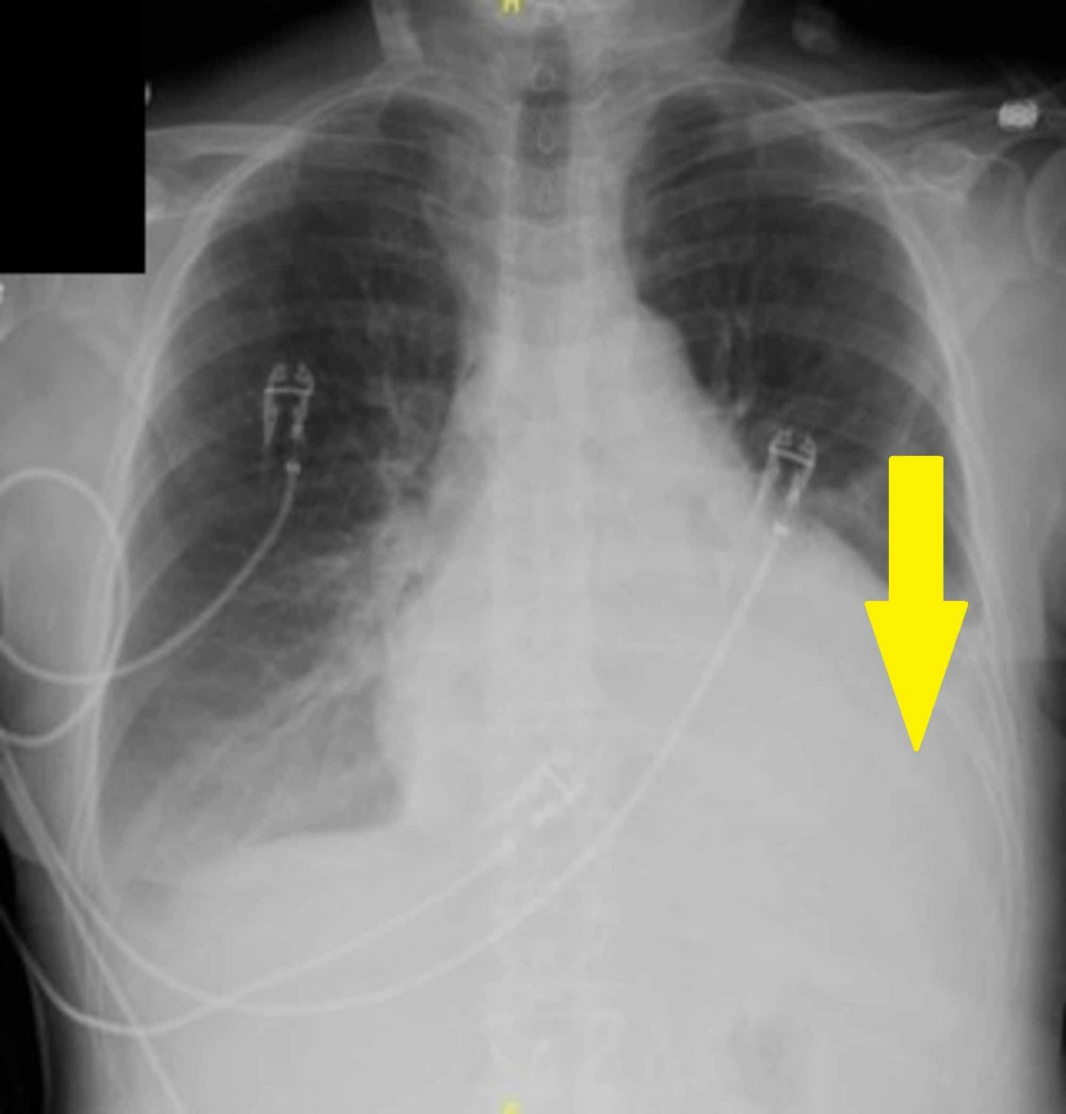
Chest Radiograph - Pleural Effusion

**Figure 2:**
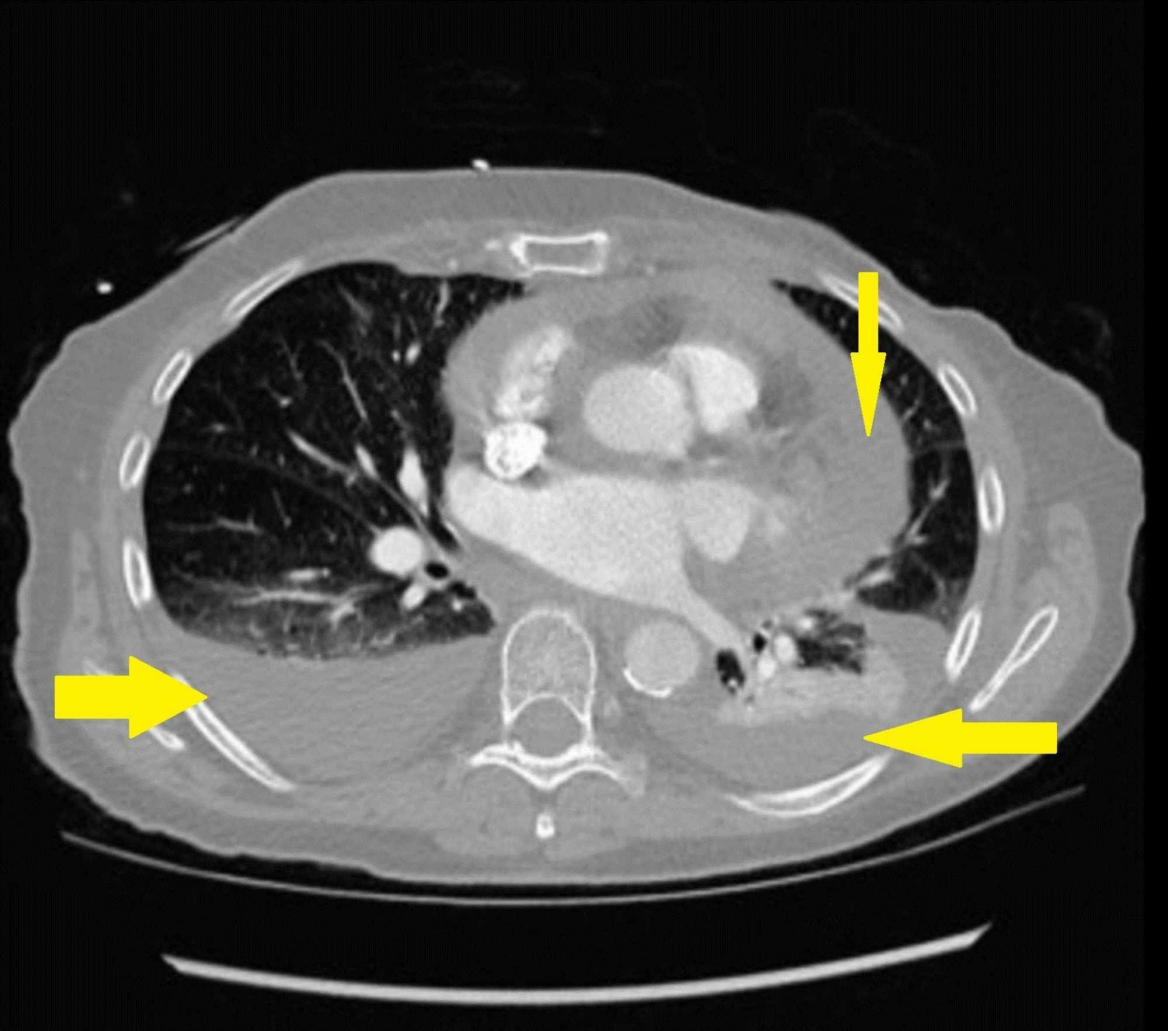
CT Chest Scan - Bilateral Pleural and Pericardial Effusions CT: Computed Tomography

A 74-year-old female presented to our hospital with complaints of involuntary weight loss, night sweats, and a one-week history of worsening dyspnea and palpitations. Her previous medical history included atrial fibrillation and mastectomy due to breast cancer; the rest of her history was otherwise unremarkable. Upon admission, she was found to be dyspneic, tachycardic, and in atrial fibrillation with a rapid ventricular response. A chest X-ray (Figure 1) followed by a computed tomography (CT) scan (Figure 2) were performed; the findings were a large pericardial effusion and moderate bilateral basal pleural effusions. Lab testing revealed anemia. Serum creatinine and calcium were within normal limits (Table 1).

**Table 1:** Laboratory Values HGB: Hemoglobin, HCT: Hematocrit, Ig: Immunoglobulin, M-spike: Monoclonal Spike

	Reference Range	Day 1	Day 45
HGB	12.0 - 16.0 g/dL	7.0 (LL)	10.2
HCT	37.0 – 47.0 %	23.4 (L)	33.3
Platelet Count	150 -400 k/uL	186	186
IgA	70 – 400 mg/dL	68 (L)	< 50 (L)
IgG	700 – 1,600 mg/dL	2,334 (H)	1,075
IgM	33 – 255 mg/dL	< 25 (L)	< 25 (L)
Kappa Light Chain	3.30 – 19.40 mg/L	705.00 (H)	11.24
Lambda Light Chain	5.70 – 26.30 mg/L	<2.10 (L)	<2.10 (L)
M Spike		1.1 mg/dL	0.5 mg/dL

**Figure 3:**
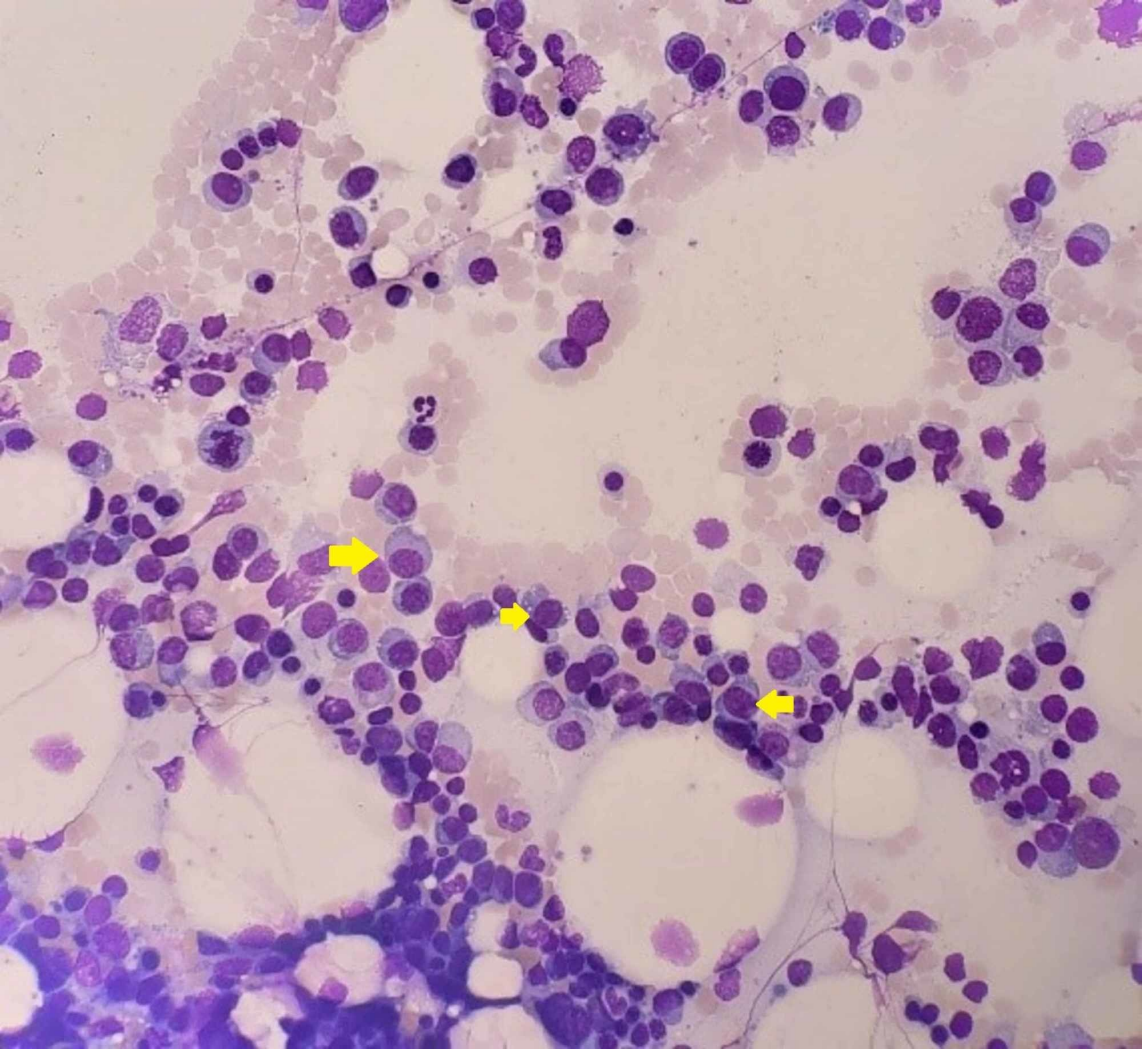
Hematoxylin and Eosin Stain of Bone Marrow Aspirate

**Figure 4:**
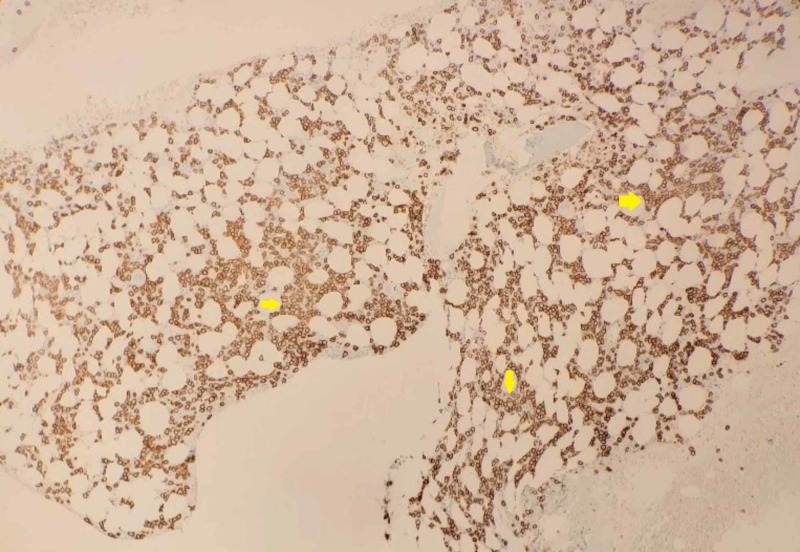
Immunohistochemistry with Anti-CD138 Stain of Bone Marrow Biopsy

The patient was admitted to the intensive care unit (ICU), where she was started on amiodarone drip and diuresis. She received three units of pRBC for symptomatic anemia. Pericardial effusion was drained, revealing 8 cc of dark fluid with no viable cells. She was investigated by hematology for a possible plasma cell dyscrasia. The free light chain ratio and immunoglobulin G (IgG) were significantly increased. A bone marrow biopsy was performed, which showed extensive plasma cell infiltration (80% involvement). Cytogenetics and fluorescence in situ hybridization (FISH) studies demonstrated a gain of chromosome 9 and 13q/14 (RB1) deletion (Figures 3-4). IgG kappa MM was diagnosed. No bone lesions were found on the staging magnetic resonance imaging (MRI) bone survey or positron emission tomography (PET) scan.

**Figure 5:**
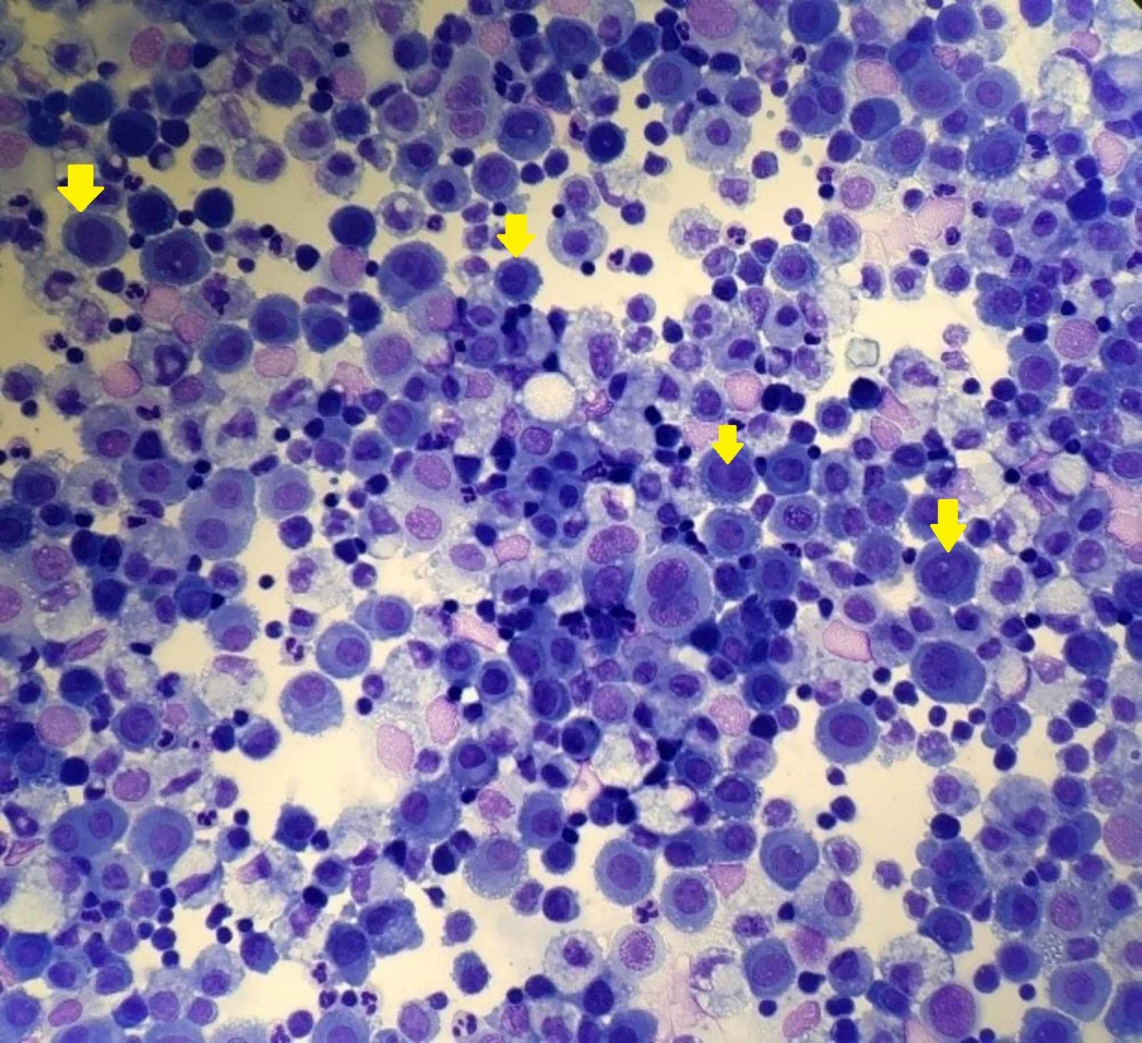
Hematoxylin and Eosin Stain of Pleural Fluid Cell Block

**Figure 6:**
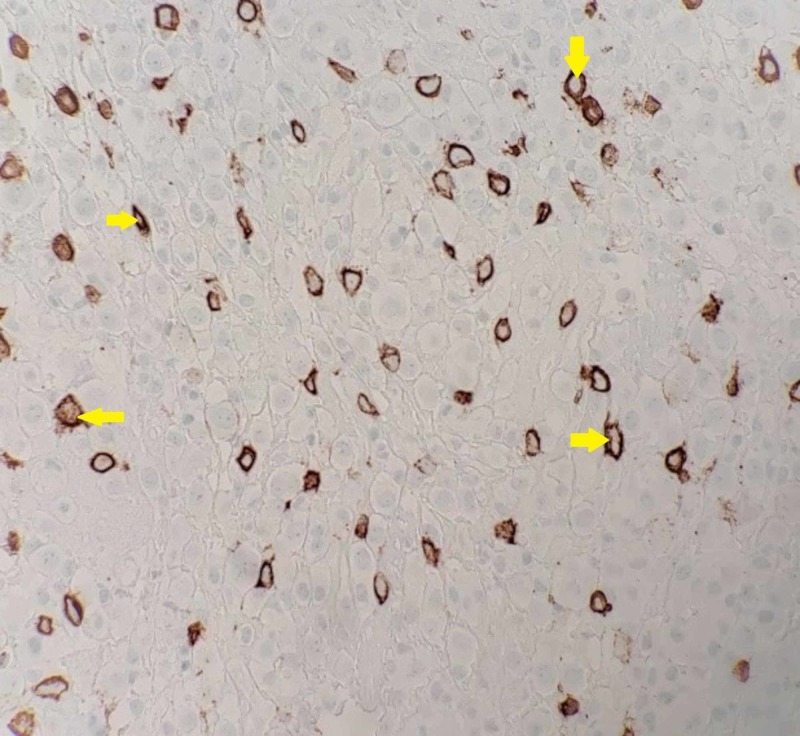
Immunohistochemistry with Anti-CD138 Stain of Pleural Fluid

Drainage of the pleural cavity collected blood-stained pleural fluid with cytologic analysis showing evidence of malignant plasma cells supporting the diagnosis of myelomatous pleural effusion (MPE) (Figures 5-6). Inpatient treatment for multiple myeloma was started with three doses of cyclophosphamide, bortezomib, and dexamethasone (CyBorD); however, rapid reaccumulation of the malignant pleural effusion required a second drainage within one week. Given the aggressive nature of her disease, treatment was switched to a bortezomib, daratumumab, and dexamethasone regimen, which induced a prompt response and resolution of the effusion and near normalization of the light chain ratio within one month. The patient is now able to perform activities of daily living independently while receiving treatment.

## Discussion

Six percent of patients with MM may present pleural effusion as a clinical symptom, with a plain range of distinct etiologies: pulmonary embolism, lymphatic drainage obstruction, chronic renal failure, and pleural myelomatous involvement; the latter represents less than 1% of all patients and is usually significant for aggressive disease and unfavorable prognoses, with a median survival time of four months [3-5]. To the best of our knowledge, concomitant pericardial effusion and MPE as initial manifestations of MM have not been reported previously.

The mechanism of disease for MPE is unknown. Local invasion from nearby bone lesions and direct infiltration as in a pleural plasmacytoma are believed to both be possible pathways for the development of this condition, most commonly presenting in later stages of MM [6].

Cytological analysis of the pleural effusion is the most common diagnostic method. A pleural biopsy may be a useful diagnostic test in excluding other etiologies (mesothelioma, extramedullary plasmacytoma) [7]. Pleural effusions in MM predominantly occur in the immunoglobulin A (IgA) subtype, up to 80% of all reported cases, thus making our case an abnormal presentation of an uncommon complication [8].

No official guideline exists for the management of MPE. Initial therapy should focus on performing a diagnostic and therapeutic thoracocentesis causing an overall respiratory symptomatic improvement. Chemotherapy treatment is usually initiated once a diagnosis has been established with variable reported results. Total regression of the MPE after only one cycle has been reported [9].

We opted for a more aggressive approach to therapy given the rapid recurrence of the MPE after an initial cycle of the CyBorD regimen, as the bortezomib, daratumumab, and dexamethasone regimen has proven to be effective in refractory MM [10].

## Conclusions

Our case demonstrates the importance of considering MPE as a possible initial presentation of MM. The development of further studies and guidelines for the management of this condition could improve the low survival rates in this patient population, especially with the recent approval of agents with high efficacy in the relapsed setting. These patients may be treated similarly as ones with poor cytogenetics given the aggressive symptomatic course and short survival. The role of high dose chemotherapy with stem cell rescue is unclear but may be considered for appropriate patients.
